# Use of the NIA LINKAGE Enclave to Study Obstructive Sleep Apnea, Cognition, and Dementia

**DOI:** 10.1093/geroni/igaf122.573

**Published:** 2025-12-31

**Authors:** Christopher Kaufmann, Marcela Blinka, Adam Spira

## Abstract

As the population ages, the number of people with Alzheimer’s disease (AD) and AD-related dementias (ADRD) will rise. While treatments to slow AD/ADRD are emerging, there is still no cure, highlighting the need to leverage data to identify risk factors for dementia prevention. The National Institute on Aging (NIA) established the LINKAGE Enclave, an online cloud-based platform facilitating access to NIA-funded cohort studies linked with administrative claims from the Centers for Medicare and Medicaid Services (CMS). Before LINKAGE, researchers had to individually apply for access from CMS, obtain permission from partner studies, and establish secure data infrastructure in their own institutions. This cumbersome process created barriers to collaboration across sites, limiting cross-site partnerships, and hindering use of these data. LINKAGE has improved data access to these unique resources. Our team, including investigators from three institutions, was among the early teams to work in the system. In our ongoing project, we are using LINKAGE to analyze two NIA-funded cohort studies with linked Medicare claims. Because our team involves multiple institutions and is working with two cohort studies, accessing and eventually using these data was not simple. We learned a tremendous amount and wish to share our experience with the aging research community. In this symposium, we present our experience using LINKAGE. We will discuss lessons learned and experiences gained, offering insights to help future teams succeed in their own projects. We encourage researchers to utilize this valuable resource, and hope it fosters research promoting dementia prevention and healthy aging.

